# Study of Bulk Amorphous and Nanocrystalline Alloys Fabricated by High-Sphericity Fe_84_Si_7_B_5_C_2_Cr_2_ Amorphous Powders at Different Spark-Plasma-Sintering Temperatures

**DOI:** 10.3390/ma15031106

**Published:** 2022-01-30

**Authors:** Yannan Dong, Jiaqi Liu, Pu Wang, Huan Zhao, Jing Pang, Xiaoyu Li, Jiaquan Zhang

**Affiliations:** 1 School of Metallurgical and Ecological Engineering, University of Science and Technology Beijing, Beijing 100083, China; ustb_dyn0801@163.com (Y.D.); 18800138678@163.com (J.L.); 2 Qingdao Yunlu Advanced Materials Technology Co., Ltd., Qingdao 266232, China; zhaohuan@yunlu.com.cn (H.Z.); pangjing@yunlu.com.cn (J.P.); fj-kjs@yunlu.com.cn (X.L.)

**Keywords:** spark plasma sintering, amorphous powders, bulk metallic glasses, sintering temperature, nanocrystalline

## Abstract

The new generation of high-frequency and high-efficiency motors has high demands on the soft magnetic properties, mechanical properties and corrosion resistance of its core materials. Bulk amorphous and nanocrystalline alloys not only meet its performance requirements but also conform to the current technical concept of integrated forming. At present, spark plasma sintering (SPS) is expected to break through the cooling-capacity limitation of traditional casting technology with high possibility to fabricate bulk metallic glasses (BMGs). In this study, Fe_84_Si_7_B_5_C_2_Cr_2_ soft magnetic amorphous powders with high sphericity were prepared by a new atomization technology, and its characteristic temperature was measured by DSC to determine the SPS temperature. The SEM, XRD, VSM and universal testing machine were used to analyze the compacts at different sintering temperatures. The results show that the powders cannot be consolidated by cold pressing (50 and 500 MPa) or SPS temperature below 753 K (glass transition temperature T_g_ = 767 K), and the tap density is only 4.46 g·cm^−3^. When SPS temperature reached above 773 K, however, the compact could be prepared smoothly, and the density, saturation magnetization, coercivity and compressive strength of the compacts increased with the elevated sintering temperature. In addition, due to superheating, crystallization occurred even when the sintering temperature was lower than 829 K (with the first crystallization onset temperature being T_x1_ = 829 K). The compact was almost completely crystallized at 813 K, resulting in a sharp increase in the coercivity of the compact from 55.55 A·m^−1^ at 793 K to 443.17 A·m^−1^. It is noted that the nanocrystals kept growing in size as the temperature increased to 833 K, which increased the coercivity remarkably but showed an enhanced saturation magnetization.

## 1. Introduction

Based on the world-class strategic goal of “energy saving, emission reduction and environmental protection”, the new integrated inductor with miniaturization, low magnetic loss and excellent stability is emerging in the electronics and electrical fields and is expected to promote the development of a new generation of high-frequency and high-efficiency motors [1,2]. Fe-based soft magnetic amorphous/nanocrystalline alloys with high saturation magnetization, low coercivity, low magnetic loss and low cost are expected to solve the problem of poor performance stability of traditional soft magnetic materials under high-frequency conditions and also provide reserve for ideal materials for integrated inductance forming [3]. At present, traditional casting technologies, such as the copper-mold-casting method and single-roll melt spinning, can be used to produce bulk metallic glasses (BMGs) with a certain thickness, but these methods are only applicable to systems with high-glass-forming ability (GFA), such as Zr, La, Pd, etc., due to the limitation of cooling rate (order of 10^2^ K·s^−1^) [4,5,6,7]. Therefore, Fe-based amorphous alloys with poor GFA are usually produced by atomization with sufficient cooling rate in industry, and then BMGs with a certain size can be fabricated by consolidation technology [8].

The traditional powder-consolidation process usually requires high-temperature conditions to produce dense bulk amorphous alloys, which cannot achieve the combination of high fraction of amorphous phase and high density. However, the development of the SPS device in 1998 made it possible to prepare BMGs with high density at low temperature [9]. With the application of an electric field and high uniaxial pressure during the SPS process, a large amount of Joule heat is generated between the powders, which improves the thermodynamic and kinetic conditions of mass transfer, and thus the powders can be consolidated into dense bulks [10]. Therefore, based on the characteristic that the viscosity of amorphous alloys decreases greatly in the supercooled liquid region, BMGs with excellent properties can be prepared by SPS within this temperature range [11]. Kim et al. prepared bulk Cu_54_Ni_6_Zr_22_Ti_18_ amorphous alloys by SPS and proposed that the strength of bulk amorphous alloys increases as the powder size decreases [12]. Li et al. prepared Al_86_Ni_6_Y_4_._5_Co_2_La_1_._5_ bulk amorphous by SPS and studied the influence of loading pressure on the densification degree and properties of bulk amorphous alloys [13]. Zrodowski et al. prepared a completely Zr-based amorphous alloy with a relative density of 100% by SPS and proved through experiments that SPS could fabricate bulk amorphous alloys with complex shapes [14]. Moravcikova-Gouvea et al. prepared Al_0_._3_NbTa_0_._8_Ti_1_._5_V_0_._2_Zr refractory by SPS and compared the influence of powder purity on the structure of BMGs by changing the milling time of the mechanical-alloying (MA) process [15]. Cheng et al. prepared Al-Zn-Mg-Cu bulk nanocrystalline materials by SPS and studied the effect of milling time on the bulk properties [16]. Gheiratmand et al. prepared FINEMET by SPS and proposed that the densification mechanism of SPS is the particle rearrangement (the first stage) and viscous flow (the second stage) [17]. In addition, for Fe-based bulk amorphous/nanocrystalline alloys, the fabrication of Fe-Si-B, Fe-Si-B-P, Fe-B-Nb-Y, Fe-Co-Ni-Zr-B, Fe-Cu-Nb-Si and Fe-Nb-Si-B systems by SPS has been reported [5,18,19,20,21].

However, most of the powders used in the above study are prepared by MA. The MA process takes a long time, and most of the powders produced have irregular shape, which can only stay in the laboratory stage, and the process has poor guiding significance for the actual industrial production of powders. In this study, the amorphous powders with high sphericity produced by the new atomization process were used as raw material, and Fe-Si-B-C-Cr BMGs with poor forming ability were prepared by SPS and cold pressing. The effects of different sintering temperatures on density, magnetic properties and mechanical properties of compacts are studied to provide practical guidance for the consolidation and performance improvement of the bulk Fe-Si-B-C-Cr system.

## 2. Materials and Methods

Commercial iron (purity > 99.9%), silicon (purity > 99.5%), boron (purity > 99.9%) and pre-alloyed Fe-B and Fe-C alloys were used as raw materials and mixed to the powder nominal composition Fe_84_Si_7_B_5_C_2_Cr_2_ (composition in wt.%). The powders were produced by the new atomization process (Qingdao Yunlu Advanced Materials Technology Co., Ltd., Qingdao, China) and then dehydrated, dried and sieved to obtain the finished products. The finished products with the same composition produced in multiple batches were mixed to reduce the quality fluctuation of a single batch of powders, and, finally, the powders used for SPS (represented by Ato) were obtained.

**Figure 1:**
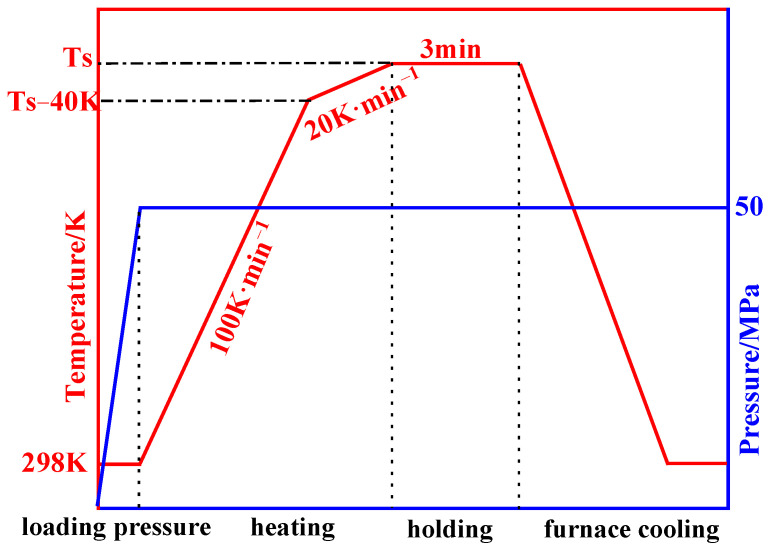
Schematic diagram of SPS heating process.

BMGs were prepared by SPS apparatus (LABOX-225, Niigata, Japan). The powders were put into a graphite die with a diameter of 12.5 mm, both ends were sealed by graphite paper and graphite punches and then the thermocouple was inserted into the central hole of the die for temperature measurement. Under the same loading pressure and holding time (fixed at 50 MPa and 3 min, respectively), different sintering temperatures were used to conduct SPS experiments. The specific heating process is shown in Figure 1. T_S_ represents the sintering temperature. A heating rate of 100 K·min^−1^ was used from room temperature to (Ts-40 K), and a heating rate of 20 K·min^−1^ was applied from (Ts-40 K) to Ts to prevent the temperature overshoot caused by a too-fast heating rate. After holding for 3 min, the furnace cooling was applied with loading the pressure. When the temperature dropped below 473 K, the pressure was unloaded. To compare with SPS, Fe-Si-B-C-Cr BMGs were fabricated by cold pressing under the pressures of 50 and 500 MPa, and the pressing time was 3 min.

The micromorphology of the atomization powders and samples by cold pressing and SPS was observed by scanning electron microscope (SEM, Phenom Pro Desktop SEM, Phenom-World BV, Eindhoven, The Netherlands). The sphericity of the atomization powders and the porosity of internal particles under different processes were calculated by the Phenom Prosuite Software (Phenom-World BV, Eindhoven, The Netherlands) and Image-Pro Plus Software (CAD/CAM Services, Inc., Celina, CA, USA), respectively. The particle-size distribution of the atomization powders was measured by the laser-diffraction particle-size analyzer (BT-9300S, Bettersize Instruments Ltd., Dandong, China). The detection particle-size range was 0.1~341 μm, the shading rate was set to 16.02%, the refractive index of the medium was set to 1.333 and the refractive index of the sample was set to 2.860. The density of compacts was measured by the Archimedean method. The amorphous structure of the atomization powders and compacts was characterized by the X-ray diffraction (XRD, D2 PHASER, BRUKER AXS, Karlsruhe, Germany) using Cu-Kα radiation with λ = 0.154184 nm. The step size was 0.02°, the scanning range was 20°~100°, the tube voltage was 30 kV and the tube current was 10 mA. The grain size of the nanocrystals was calculated by Jade 9.0 software (Materials Data, Inc., Livermore, CA, USA). The characteristic temperature and the crystallization enthalpy of the atomization powders and compacts at different sintering temperatures were measured by the differential scanning calorimeter (DSC, Setaram Setsys Evo, KEP Technologies, Beijing, China) at a heating rate of 10 K·min^−1^ and a 30 mL·min^−1^ flow rate of high-purity argon. The saturation magnetization and coercivity of the atomization powders and compacts were measured by the vibrating-sample magnetometer (VSM, Lake Shore 8604, Lake Shore Cryotronics, Inc., Westerville, OH, USA) at the maximum applied magnetic-field intensities of ±10 and ±200 Oe, respectively. The compressive strength of the compacts was measured by the microcomputer-controlled universal materials-testing machine (WDW-10E, Jinan shidai shijin Testing machine Group Co., Ltd., Jinan, China) at a compression rate of 0.1 mm·min^−1^ and room temperature of 298 K. One sample prepared under each sintering condition was used for the compression test, with a size of φ 12.5 mm × 8 mm.

## 3. Results

### 3.1. Characterization of Atomization Powders

**Figure 2:**
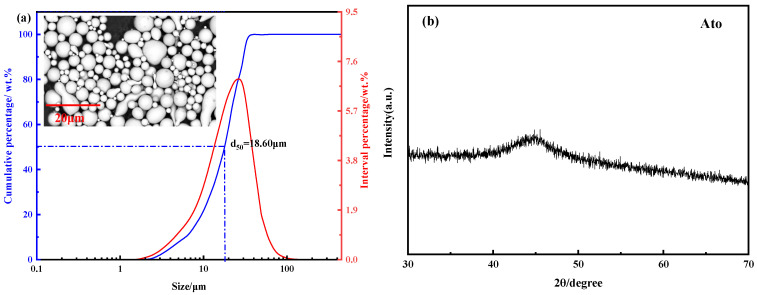
Characterization of atomization powders: (**a**) SEM image and the size distribution and (**b**) XRD pattern.

Figure 2a displays the morphology of Fe_84_Si_7_B_5_C_2_Cr_2_ atomization powders and the particle-size distribution measured by the laser-diffraction particle-size analyzer. As can be seen from the figure, the surface morphology of the powders was smooth, and the sphericity of the powders was calculated to be 0.986 by the Phenom Prosuite Software. Compared with MA powders, the atomization powders used in this study had highly regular shape and sphericity characteristics [22]. The d_50_ of the powders was 18.60 μm, and the particle-size distribution was similar to the normal distribution, and the distribution range was narrow, with very few large particles over 100 μm. The XRD pattern of the atomization powders is shown in Figure 2b. The XRD image shows an obvious broad peak, indicating that the powders with a diameter of less than 100 μm prepared by the new atomization process were completely amorphous.

**Figure 3:**
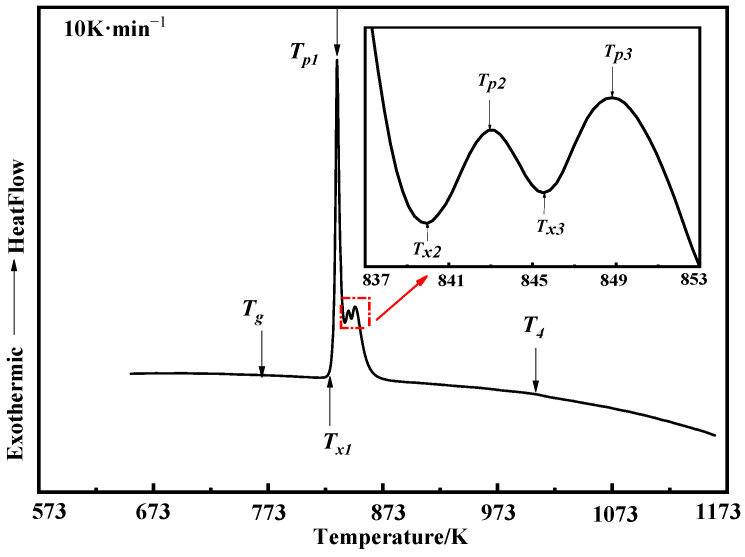
The DSC curve of the Fe_84_Si_7_B_5_C_2_Cr_2_ atomization powders (10 K·min^−1^).

The DSC curve of the Fe_84_Si_7_B_5_C_2_Cr_2_ atomization powders at the heating rate of 10 K·min^−1^ is shown in Figure 3, and the characteristic temperature and crystallization enthalpy are listed in Table 1. The DSC curve shows that there were three obvious crystallization peaks in the amorphous system during heating. The crystallization enthalpy ΔH_1~3_ of the first three crystallization peaks was 120.68 J·g^−1^. According to the study by Ustinovshikov et al. [23], the fourth phase transition that occurred when T_4_ = 1004 K corresponded to the second-order phase transition that occurs in the Fe-Si alloy system at temperatures above 973 K, so there is no exothermic peak in the DSC curve. Noting the glass transition temperature T_g_ of the atomization powders was 767 K and the primary crystallization temperature T_x1_ was 829 K, the undercooled-liquid region ΔT = T_x1_ − T_g_ = 62 K was obtained. The wide undercooled-liquid region and high-crystallization temperature indicate that the Fe-Si-B-C-Cr amorphous system has excellent processing performance and thermal stability, which is beneficial for choosing the appropriate temperature for SPS.

### 3.2. Densification Behavior

**Figure 4:**
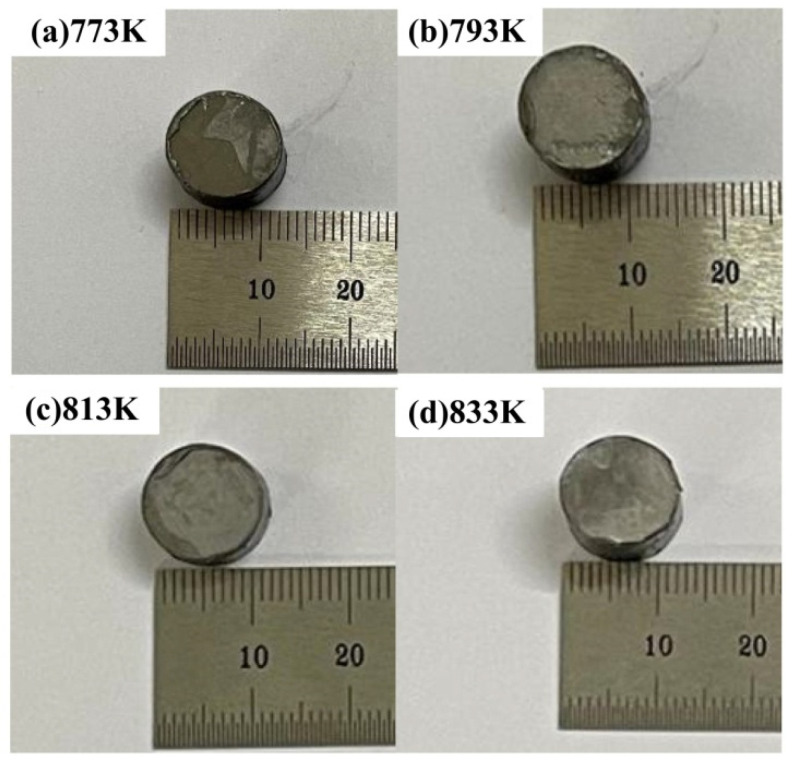
Macro-morphology of compacts by SPS: (**a**) 773 K, (**b**) 793 K, (**c**) 813 K and (**d**) 833 K.

The bulk sample could not be fabricated by cold pressing and SPS at 523 K or 673 K. Although the bulk was prepared successfully at 753 K, it broke after removing it from the graphite die due to the poor strength. Therefore, Figure 4 presents the compacts prepared by SPS at 773 K, 793 K, 813 K and 833 K. The average size of the samples was φ 12.5 mm × 8 mm.

**Figure 5:**
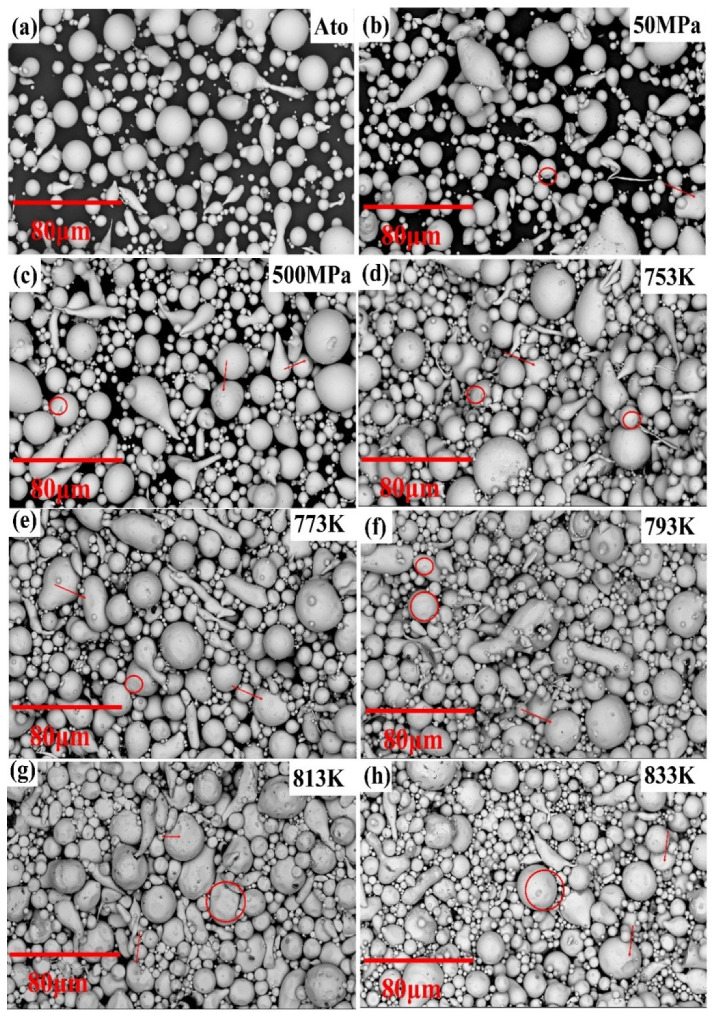
SEM micrographs for (**a**) atomization powders, (**b**,**c**) samples by cold pressing at 50 MPa and 500 MPa, respectively, and (**d**–**h**) SPS samples at different sintering temperatures.

Figure 5 gives the SEM images of samples under different processes. The raw powder particles with good sphericity had obvious indentation after cold pressing, while compacts prepared by SPS had plastic-smooth indentation. Table 2 summarizes the density of samples prepared by different processes and the porosity calculated by Image-Pro Plus Software (black area/total area × 100%). The theoretical density of Fe_84_Si_7_B_5_C_2_Cr_2_ powders is 5.80 g·cm^−3^. The original atomized-powder density is 4.46 g·cm^−3^, and its porosity is 42.4%. The results show the consolidation ability of the cold-pressing process was poor. Even if the pressure was increased to 500 MPa, a dense bulk amorphous alloy could not have been prepared, and the powder porosity was only reduced by 9.6% compared to the raw powders. In contrast, the SPS can significantly improve the density of samples. As the sintering temperature increased from 753 K to 833 K, the sample porosity decreased from 23.7% to 9.7%, which was consistent with the relationship between density and sintering temperature proposed by Chen et al. [24] based on the viscous flow during sintering.

### 3.3. Crystallization Behavior

**Figure 6:**
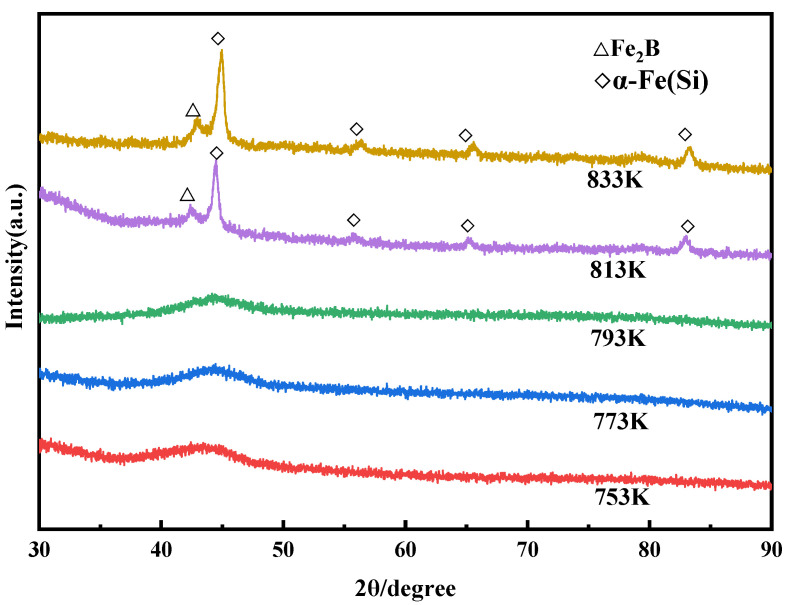
XRD patterns of compacts at different sintering temperatures by SPS.

The XRD pattern of compacts at different sintering temperatures is plotted in Figure 6. There is no typical sharp peak in the sintering temperature range of 480~793 K, and the pattern still shows an obvious broad-halo diffuse-scattering wide peak. Even at 793 K above T_g_, the bulk remained mainly amorphous. However, with the increase in sintering temperature to 813 K and 833 K, the diffraction peak of the amorphous compacts in the range of 43°~45° becomes narrow, the FWHW (full width of half height) becomes smaller and more sharp diffraction peaks appear, which correspond to the crystal phases of Fe_2_B and α-Fe(Si). Based on the Scherrer formula and Jade software, the average grain sizes of Fe_2_B and α-Fe(Si) phases are 14.42 nm and 15.62 nm at 813 K, respectively, and increase to 15.65 nm and 18.05 nm at 833 K, respectively, which indicates that the nanocrystalline phase grew slowly with the increase in sintering temperature. In addition, sharp peaks appear at 813 K below T_x1_ = 829 K, which is similar to the results from Feng et al. [5]. In order to quantitatively analyze the amorphous fraction of compacts more accurately, DSC was conducted for compacts at different sintering temperatures.

**Figure 7:**
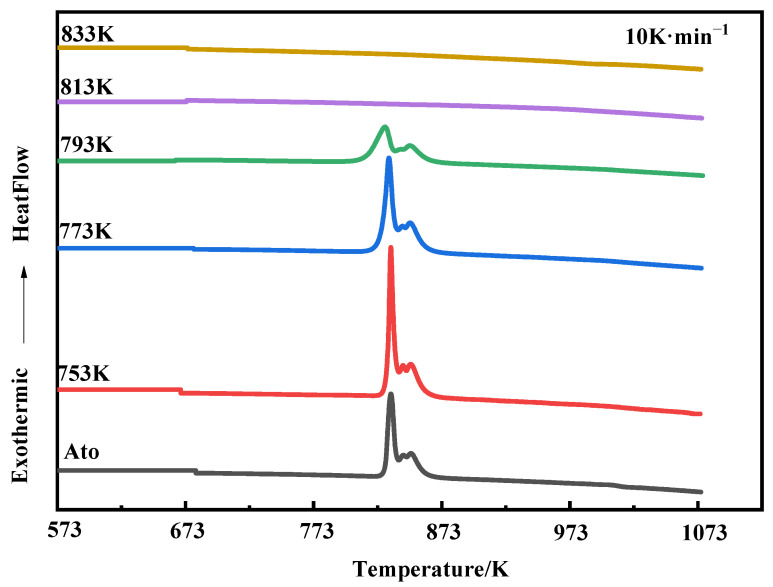
DSC curves of the atomization powders and compacts at different sintering temperatures (10 K·min^−1^).

Figure 7 compares the DSC curves of the atomization powders and compacts at a heating rate of 10 K·min^−1^. The crystallization enthalpy corresponding to the crystallization peak can be obtained through data processing. The crystallization enthalpy represents the energy released by the amorphous compacts during crystallization. For the amorphous system with the same composition, the higher the crystallization enthalpy measured under the same conditions, the higher the fraction of amorphous phase. Therefore, the amorphous fraction of the compacts can be calculated by Equation (1) [25]:

(1) $$X_{a}=\frac{\Delta H}{\Delta H_{max}}\times 100\%,$$

where $X_{a}$ is the fraction of amorphous compact, pct.; ΔH is the crystallization enthalpy of the compacts, J·g^−1^ and $\Delta H_{max}$ is the crystallization enthalpy of the completely amorphous sample, that is, enthalpy of crystallization of the atomization powders, J·g^−1^.

According to Table 1, since the fourth phase transition belongs to the second-order phase transition, $\Delta H_{max}\approx \Delta H_{1\sim 3}$ could be defined, and ΔH could also be approximated as the first three crystallization enthalpies. When the sintering temperature is 753, 773, 793, 813 and 833 K, the corresponding crystallization enthalpies are 119.784, 116.133, 113.084, 0 and 0 J·g^−1^, respectively. The fractions of amorphous compacts are 99.3%, 96.2%, 93.7%, 0% and 0%, respectively, which is also consistent with the qualitative analysis of XRD, that is, the samples are mainly amorphous when the sintering temperature is not higher than 793 K, while when the sintering temperature increases to 813 K, the bulk becomes completely crystallized. In addition, nanocrystal precipitation is also the main reason for the rapid increase in the density of the compacts from 793 to 813 K during the densification behavior mentioned above.

Based on other studies on crystallization during SPS [5,14,22,26], the reasons for the occurrence of crystallization in the T_x1_ in this study are as follows:

(1) Unlike conventional heating methods, SPS generates Joule heat directly inside the compacts, which can result in the graphite-die temperature measured by the thermocouple being lower than the actual internal temperature of the compacts.
(2) The electrical resistance in the particle-contact area is large, generating a large amount of Joule heat and local overheating.
(3) The crystallization process is a kinetic behavior related to the heating rate, and the characteristic temperature of the amorphous system increases with the increasing of the heating rate. Therefore, the actual crystallization temperature during the holding progress of SPS may be less than T_x1_ measured at the heating rate of 10 K·min^−1^.

### 3.4. Magnetic Properties and Mechanical Properties

**Figure 8:**
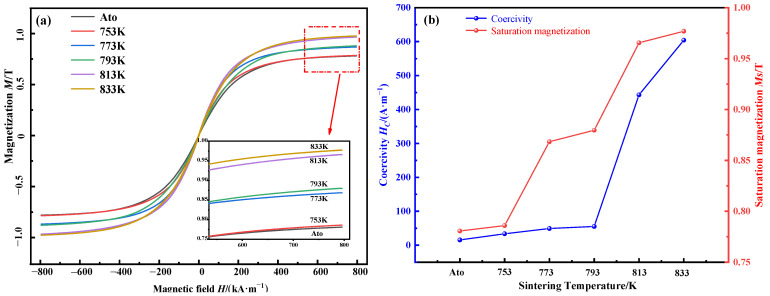
The raw powders and compacts at different sintering temperatures for (**a**) hysteresis loops and (**b**) magnetic properties.

Figure 8a,b illustrate the hysteresis loops and magnetic properties of the compacts at different sintering temperatures and of the atomization powders. It can be seen from the figure that the saturation magnetization (*M*_s_) of SPS samples was higher than that of the atomization powders, and the *M*_s_ increased from 0.79 T at 753 K to 0.98 T at 833 K. With the increase in sintering temperature, the porosity in the compacts decreases and the density increases, resulting in the increase of the content of effective magnetic phase in the compacts. On the other hand, the precipitation of α-Fe(Si) nanocrystalline phase with ferromagnetism enhances the overall ferromagnetic-coupling effect and improves the *M*_s_ of the compacts with the increase of temperature to 813 K. However, the coercivity (*H*_c_) of SPS samples was higher than that of the atomization powders, and the *H*_c_ of the compacts at 793 K was 39.87 A·m^−1^ higher than that of the atomization powders. Moreover, the *H_c_* increased rapidly from 55.55 A·m^−1^ at 793 K to 443.17 A·m^−1^ at 813 K and to 604.39 A·m^−1^ at 833 K. This is because coercivity is a physical quantity sensitive to structure, and factors such as the grain shape, crystal defects, stress distribution, second phase distribution and grain size will have a great influence on it [27]. The heating rate during SPS is very fast, which inevitably leads to stress concentration in the compacts, thus enhancing the magnetocrystalline anisotropy of soft magnetic phase and increasing the *H*_c_. On the other hand, the oxidation and residual graphite impurities, air gaps and crystals with large size difference during SPS will hinder the movement of magnetic domain walls and increase the *H*_c_ [5]. When the temperature increased to 813 K, the soft magnetic amorphous phase in the compacts completely crystallized, resulting in the sudden rise of the *H*_c_ at sintering temperature above 793 K. When the sintering temperature continued to increase from 813 to 833 K, the increase in *H*_c_ could be explained by the ferromagnetic-exchange-coupling model of nanocrystalline alloys proposed by Herzer for Fe-based nanocrystalline alloys [28], that is, for nanocrystals with sizes smaller than the ferromagnetic exchange length. The *H*_c_ increases with the growth of nanocrystals, which is consistent with the grain size calculated above.

**Figure 9:**
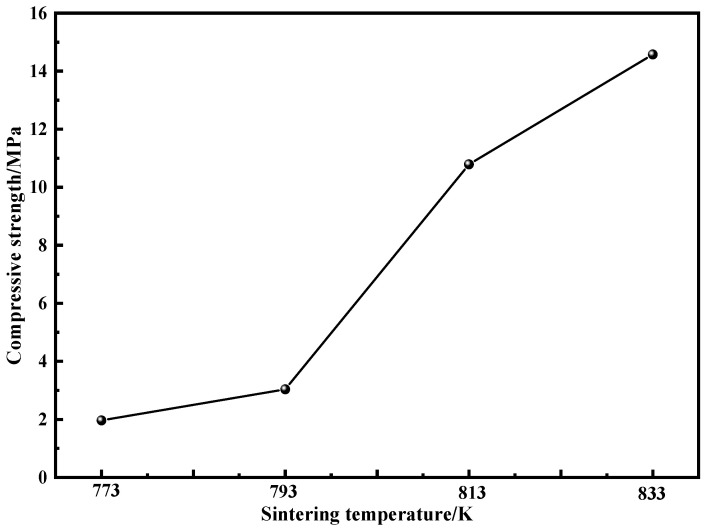
Compressive strength of compacts at different sintering temperatures.

Figure 9 shows the results of compressive strength at different sintering temperatures. The density of the compacts increases with the increase in sintering temperature, which leads to the increase in the compressive strength, and the compressive strength can be increased to 10.8 MPa at the sintering temperature above 813 K due to the precipitation of a large number of fine nanocrystals. However, a small amount of powder falling from the compacts was observed during the process of compression testing, which indicated that the internal bonding force between particles of the compacts was still unsatisfactory. The main reason is that the loading pressure is too small due to the limitation of graphite-die size and experimental equipment parameters. Li et al. [13] proposed that the loading pressure, compared with the sintering temperature, could improve the density of the compacts more effectively and avoid the deterioration of mechanical properties by coarse nanocrystals. Therefore, the authors will continue to study the loading pressure for the fabrication of Fe-Si-B-C-Cr bulk amorphous/nanocrystalline alloys after this work.

## 4. Conclusions

In this paper, Fe_84_Si_7_B_5_C_2_Cr_2_ industrial atomization amorphous powders with high sphericity are used as raw material to fabricate bulk amorphous/nanocrystalline alloys by cold pressing and SPS technology. The effects of sintering temperature on the densification behavior, crystallization, magnetic properties and mechanical properties of the compacts are studied. The main conclusions are as follows:

- The glass transition temperature (T_g_) and the first crystallization onset temperature (T_x1_) of Fe_84_Si_7_B_5_C_2_Cr_2_ amorphous powders produced by the new atomization process at the heating rate of 10 K·min^−1^ are 767 K and 829 K, respectively, and the undercooled-liquid region (Δ*T*) is 62 K, which indicates excellent processing performance and thermal stability of the amorphous system.
- The bulk metallic glasses (BMGs) cannot be prepared by cold pressing of 50 and 500 MPa or SPS temperature below 773 K. The porosity of the compacts decreases with the increase in temperature, which promotes the densification, indicating the superiority of SPS technology over traditional powder-consolidation technology.
- The crystallization enthalpy and the amorphous fraction of the compacts decrease with the increase in sintering temperature, and the Fe_2_B and α-Fe(Si) nanocrystals precipitate at 813 K, which makes the compacts almost completely crystallize, even below T_x1_. The precipitated nanocrystals also grow with the increase in temperature, and they can further improve the density by filling the pores.
- The saturation magnetization (*M*_s_) of the compacts increases with the increase in sintering temperature due to the increase in the content of effective magnetic phase and the precipitation of the ferromagnetic α-Fe(Si). As the sintering temperature increases, the coercivity (*H*_c_) increases due to the internal stress and the precipitation of nanocrystals. The increase in relative density and the pinching effect of fine nanocrystals can strengthen the compressive strength.

In this study, although the Fe-Si-B-C-Cr bulk amorphous/nanocrystalline alloys with excellent soft magnetic properties can be prepared by adjusting the sintering temperature, the mechanical properties are still unsatisfactory, which should be caused by the limited load presently. The following work will focus on improving the loading pressure to fabricate the Fe-Si-B-C-Cr compacts with excellent soft magnetic properties together with an improved mechanical strength.

## Figures and Tables

**Table 1:** Characteristic temperature and crystallization enthalpy of the Fe_84_Si_7_B_5_C_2_Cr_2_ atomization powders (10 K·min^−1^).

T_g_	T_x1_	T_p1_	T_x2_	T_p2_	T_x3_	T_p3_	ΔH_1~3_	T_4_
767 K	829 K	834 K	840 K	843 K	846 K	850 K	120.68 J·g^−1^	1004 K

**Table 2:** Density and porosity of samples prepared by different processes.

Process	Temperature/K	Pressure/MPa	Forming	Density/(g·cm^−3^)	Relative Density	Porosity
Cold pressing	298	50	×	/	/	34.8%
	298	500	×	/	/	32.8%
SPS	523	50	×	/	/	/
	673	50	×	/	/	/
	753	50	×	/	/	26.7%
	773	50	√	4.79	82.6%	23.7%
	793	50	√	4.98	85.9%	17.6%
	813	50	√	5.54	95.5%	11.5%
	833	50	√	5.60	96.6%	9.7%

## Data Availability

The data presented in this study are available on request from the corresponding author.
